# Low appendicular muscle mass is correlated with femoral neck bone mineral density loss in postmenopausal women

**DOI:** 10.1186/1471-2474-12-225

**Published:** 2011-10-07

**Authors:** Fábio L Orsatti, Eliana AP Nahas, Jorge Nahas-Neto, Cláudio L Orsatti, Moacir Marocolo, Octávio Barbosa-Neto, Gustavo R da Mota

**Affiliations:** 1 Exercise Biology Laboratory (BioEx), Health Sciences Institute, Triângulo Mineiro Federal University (UFTM), Uberaba-MG, Brazil; 2 Department of Gynecology and Obstetrics, Botucatu Medical School, São Paulo State University- UNESP, Brazil; 3 Post-graduation Program in Gynecology, Obstetrics and Mastology, Botucatu Medical School, São Paulo State University- UNESP, Brazil

**Keywords:** sarcopenia, osteoporosis, menopause, body composition, DXA

## Abstract

**Background:**

After menopause, rapid bone mass loss occurs in response to hypoestrogenism. Several studies suggest that muscle mass and bone mineral density (BMD) are positively associated in postmenopausal women. Therefore, it may be assumed that postmenopausal low appendicular muscle mass (aMM) can increase BMD loss in a short period of time.

**Objective:**

The purpose of this study was to assess relationship of aMM with femoral neck BMD in postmenopausal women.

**Methods:**

Prospective, controlled clinical Trial including 64 women aged 45-70 years, who had not had their last menstruation for at least one year. Subjects were divided into two groups: low aMM (n = 32), and normal aMM (n-32). Femoral neck BMD and muscle mass were measured by DXA at baseline and after twelve months. Pairwise and independent t tests were used for data analysis.

**Results:**

Baseline weight, BMI and muscle mass (total and appendicular) significantly differ between groups (p < 0.05). After twelve months, femoral neck BMD was significantly lower in the group with low aMM, whereas no significant difference was observed in the group with normal aMM (p < 0.05).

**Conclusion:**

In postmenopausal women, low appendicular muscle mass is associated negatively with femoral neck BMD in a short period of time.

## Background

During the first years after menopause, rapid bone loss occurs in response to hypoestrogenism [1]. The decline in bone mineral density (BMD), and structural integrity increase the risk of osteoporosis in postmenopausal women [2]. The most common clinical consequence of osteoporosis is fracture, especially of the femoral neck, vertebrae and wrist [2], that lead to functional impairment [3]. In 2005, osteoporosis-related fractures cost 17 billion of dollars to the United States. Hip fractures accounted for 14% of all fractures and 72% of the costs [4].

BMD is influenced by several factors. In postmenopausal women, muscle mass (MM) and body weight has been positively associated with femoral neck BMD [5-9]. Transition into menopause has been associated with MM reduction [10,11]. High MM loss, together with loss of muscle strength and/or function is named sarcopenia [12]. Some investigators have proposed measuring the muscle mass of the four limbs (appendicular muscle mass) by DXA (dual-energy X-ray absorptiometry) to determine low muscle mass [12,13]. Defining muscle mass index as aMM/height^2^, a muscle mass index two standard deviations (2SD) below the mean muscle mass index of gender-specific reference groups of young adults indicates low muscle mass [12,13].

Several hypotheses have been built to explain the association of MM on bone mass. Muscle strength gains are believed to induce periosteal aposition directly stimulating, via mechanic strength, osteocyte mechanoreceptors [14,15]. Additionally, bone and muscle share endocrine and genetic influences. Muscle has an endocrine function by producing bioactive molecules that can contribute to homeostatic regulation of both bone and muscle [14,15]. Bone and muscle also share genetic determinants. Therefore, the consideration of pleiotropy is an important aspect in the study of the genetics of osteoporosis and sarcopenia [14].

Considering muscle mass as an indicator of BMD, It can be postulated that low muscle mass in postmenopausal women could influence the rate of bone loss. Thus, the purpose of this study was to assess femoral neck BMD in postmenopausal women with or without low aMM.

## Methods

### Study design and sample selection

A prospective, controlled study with pre- and post-test assessments was conducted. The study population consisted of women attending the Climacterium & Menopause Service of Botucatu Medical School, São Paulo State University-UNESP. The study participants were healthy women aged 45-70 years that had not had their last menstruation at least 12 months prior to the study, and FSH (follicle-stimulating hormone) levels greater than 40 mIU/ml. Exclusion criteria were: (1) hormone replacement therapy; (2) history of myopathy, neuropathy, or skeletal disease; (3) history of catabolism-elevating diseases such as cancer, nephropathies and hepatopathies; (4) alcoholism; (5) chronic gastrointestinal disease; (6) athletes; (7) use of medication known to have metabolic effects on bone and muscle.

Of all the women attending our service, 84 were identified as potentially eligible. They were informed about the study objectives and procedures, and asked to provide their written consent to participate. The study was approved by the local Committee of Research Ethics.

Initially, all subjects underwent history taking, physical and gynecological examination and body composition evaluation, as well as femoral neck BMD measurement by DXA. Data collected included age, aMM, body fat, %fat, and femoral neck BMD. After screening was completed, 64 women were enrolled and allocated into two groups of 32 easch: 1) low aMM; and, 2) normal aMM [13]. All 64 subjects were followed up for 12 months with femoral neck BMD being measured at baseline and at the end of the study.

### Anthropometric assessment

Weight was measured using a platform balance beam scales (150 kg capacity, 100 g divisions, 0.1 kg precision; Filizola^®^, Brasil) with women wearing no shoes and light clothes. Height was determined using a portable stadiometer (0.1 cm precision; Seca^®^, Brasil) fixed on wall. Body mass index (BMI) was classified according to the system used by the World Health Organization (2002): < 18.5 kg/m^2 ^= underweight; 18.5 - 24.9 kg/m^2 ^= normal weight; 25 - 29.9 kg/m^2 ^= overweight; 30.0 - 34.9 kg/m^2 ^= obese class I; 35.0 to 39.9 kg/m^2 ^= obese class II; ≥ 40.0 kg/m^2 ^= obese class III.

### Bone mineral density and body composition

**Figure 1:**
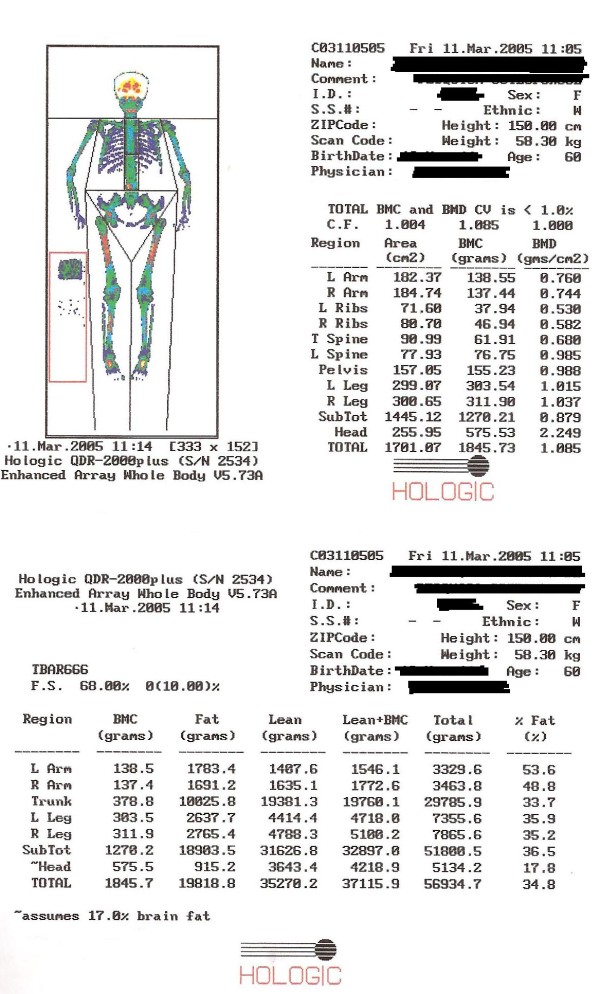
**Representative DXA images with fat/bone/muscle segmentations illustrated for various body regions**.

Femoral neck BMD and body composition (fat mass and fat-and bone-free mass) were measured by dual-energy X-ray absorptiometry (DXA), at baseline and 12 months later, using a Hologic QDR-2000 densitometer (Hologic^®^, Waltham, MA, USA). Patients were instructed to remove metal objects (e.g., snaps, belts, underwire bras, jewelry) and their shoes and were dressed only in a hospital gown. Patients lay supine with their arms at their sides and were instructed to remain motionless during the scan. To minimize interobserver variation, all scans and tests were performed by the same certified densitometry technologist. Intravariation in femoral neck BMD assessment was 1%. BMD was reported in g/cm^2^. Body composition was determined using the manual analysis software (version 4.76A:1 for BMD and 5.73A). The arm region was defined as the region extending from the head of the humerus to the distal tip of the fingers. The reference point between the head of the humerus and the scapula was set at the glenoid fossa. The leg region was defined as the region extending from the inferior border of the ischial tuberosity to the distal tip of the toes (Figure 1). The appendicular muscle mass (aMM) was defined as the summation of the muscle mass (fat-and bone-free mass) of the four limbs (arms and legs). Total fat mass and fat-and bone-free mass (tMM) were defined as the region extending from the shoulders to the distal tip of the toes. In-house CV on a subsample of women is, <1.0 - 3.0% for body composition measures [13,16,17]. Data from our laboratory showed that the coefficient of variation for body composition measures was < 1.0% [16]. Measurements of muscle mass by DXA have been validated [18] against multislice computed tomography of the legs in 60 persons aged 70-79 (R^2 ^= 0.96, SEE = 0.7 kg)[19] and against MRI of the legs in 101 postmenopausal women similar to our study aged 70.7 ± 6.4 y and their mean BMI was 27.4 ± 5.1 kg/m2 (R^2 ^= 0.82, RMSE = 0.82 kg)[20].

Low aMM was defined as 2 SD below the mean for gender-specific reference group of young adults as described by Baumgartner et al.[13]. A muscle mass index was obtained by dividing aMM (summation of the muscle mass of the four limbs) by the squared height (m^2^). The cut point used was that proposed by Baumgartner et al.[13] for women (<5.45 kg/m^2^)

### Statistical analysis

Statistical analysis was performed using the statistical software for Windows. Variable distribution was assessed by the tests of Shapiro-Wilk, Kolmogorov & Smirnov. A log transformation was applied to the variables not normally distributed (FSH and E_2_). Normally distributed variables were reported as mean and standard deviations. Differences between groups in baseline characteristics were tested using a t unpaired test. A pairwise t test was employed to test the effect of time (pre vs posttesting). The relationship between baseline variables and femoral neck BMD changes was determined by Pearson's correlation (delta = pretesting value - posttesting value). Significance level was set at 5% (p < 0.05).

## Results

**Figure 2:**
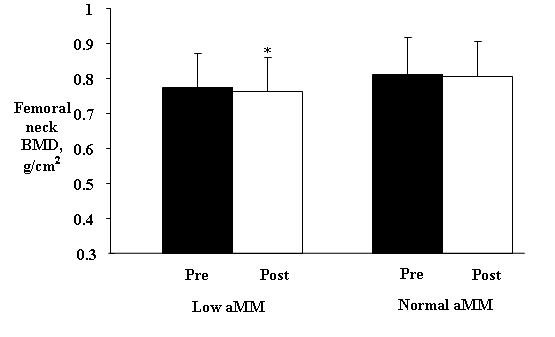
**Effect of twelve months on femoral neck BMD in women with or without low aMM (mean ± SD)**. *pairwise *t *test, significant difference p < 0.05.

In this study, group assignment was based on appendicular muscle mass. Thus, as expected, no significant differences between groups were observed in baseline parameters, except weight, BMI, and muscle mass (total and appendicular) (Table 1). After 12 months, femoral neck BMD was significantly lower in the group with low aMM (Low aMM: pre = 0.775 ± 0.095, post = 0.764 ± 0.096 and P = 0.002), whereas the group with normal muscle mass showed no significant differences (Normal aMM: pre = 0.812 ± 0.104, post = 0.807 ± 0.098 and p = 0.368) (Figure 2).

**Table 1:** Anthropometric and demographic characteristics of the subjects in the study at baseline

*Variables*	*Low aMM (n = 32)*		*Normal aMM (n = 32)*		
	Mean	SD	Mean	SD	*p *value^*^
Age (years)	57.0	7.4	55.4	7.6	0.397
Time since menopause (years)	7.6	5.8	8.3	5.7	0.618
Weight (kg)	65.9	10.9	76.5	12.9	0.001
BMI (kg/m^2^)	26.7	3.7	31.7	4.7	<0.001
Total MM (kg)	30.3	3.2	35.9	4.2	<0.001
Appendicular MM (kg)	11.9	1.4	14.6	1.9	<0.001
Appendicular MM/m^2 ^(kg/m^2^)	4.8	0.5	6.1	0.5	<0.001
Total fat (kg)	26.7	7.8	32.6	9.2	0.007
% fat	45.2	5.4	45.44	5.9	0.874
logFSH	4.2	0.3	4.1	0.3	0.334
logE2	3.0	0.2	3.1	0.2	0.100

SD, standard deviation.

*unpaired t test, significant difference p < 0.05

Femoral neck BMD change over 12 months (Δ delta = posttesting - prettesting) correlated significantly only with baseline BMI, aMM and aMM/height2 values (Table 2). BMI showed significant positive correlations with aMM and aMM/m2 (r = 0.66 and r = 0.73, respectively).

**Table 2:** Correlations between baseline parameters and femoral neck BMD changes after twelve months (Δ delta = pre - post) in 64 postmenopausal women

	Age	TM	Weight	BMI	tMM	aMM	**aMM/m**^**2**^	Fat	% fat
Δ Femoral neck BMD	-0.09	-0.08	0.19	0.26	0.24	0.26	0.27	0.15	0.08
P value	0.472	0.508	0.122	0.042	0.057	0.038	0.029	0.223	0.554

TM, time since menopause; BMI, body mass index; aMM, appendicular muscle mass; aMM/m^2^, mucle mass index.

## Discussion

This prospective controlled trial tested the hypothesis that low aMM is associated with loss of femoral neck BMD over a short period of time in postmenopausal women. On the basis of the cut point proposed by Baumgartner et al [13], femoral neck BMD significantly decreased in postmenopausal women with low aMM, but not in those with normal aMM, during a period of 12 months. This finding is in agreement with cross-sectional studies reporting association between muscle mass and femoral neck BMD [5-7,21].

Our results show that, in relation to time, the effect of aMM and aMM/height^2 ^(muscle mass index) on femoral neck BMD is stronger than that on the other anthropometric parameters assessed. Di Monaco et *al*. (2010), demonstrated that aMM/height^2 ^had a significant positive correlation with femoral neck BMD in 313 elderly women. As a consequence, the odds ratio for osteoporosis in women with low aMM was 1.8 (95%CI = 1.073-3.018) [5].

The reason why aMM and aMM/height^2 ^correlated more strongly with femoral neck BMD than total muscle mass may be the fact that DXA-derived measurement includes lean trunk organ tissues, a great amount of skin and nonfat components in the adipose tissue. Chen et al. (2007) reported a ratio of MM (DXA):MM (MRI) was only 1.5 for the leg region, but increased to 2.1 for the total body [20].

Bone has the ability to sense loading-induced deformation and to adapt its structure to the load it receives. All body movements are produced by coordinated contractions of the striated muscle, while the associated muscle work comprises the fundamental source of mechanical loading to the skeleton positively acting on bone mass. This is the theoretical support for the association of muscle mass on bone mineral density [22]. In addition, muscle-released bioactive molecules and genetic factors can also contribute to regulation of both bone and muscle [14].

Although the relationship between muscle strength and muscle mass is not linear, age-related muscle loss may progressively decrease strength and power (force vs velocity), impairing the ability to perform activities of daily living [12]. This significantly reduces the mechanical load to the skeleton, contributing for the development of bone frailty and functional decline at advanced age. Thus, dual-energy X-ray absorptiometry screening should be used to identify both BMD and muscle mass in postmenopausal women to assess more accurately the risk of fractures and disability, as suggested by Gentil et al [6]. The beneficial effect of resistance training on muscle mass, muscle strength and BMD in postmenopausal women has been well established [23-25]. Besides producing direct effects on BMD, resistance training has been associated with decrease in functional disability, as well as in the occurrence of falls and fractures [25]. Therefore, resistance training has been widely indicated for the prevention or treatment of sarcopenia and osteoporotic fractures [3].

Weight loss significantly increases postmenopausal bone loss [8,9,26], but the effects of muscle mass change on weight-loss-associated bone loss remain unclear. Sirola et al., showed that maintaining muscle strength may counteract postmenopausal bone loss related to weight loss [27]. In this study, a significant positive correlation was observed between BMI and femoral neck BMD. However, such effect might have been mediated by muscle mass as correlations with body weight and fat were not significant. Other investigators did not find any association between body fat and BMD either [7,28]. Indeed, women tend to accumulate visceral fat with menopause. This appears to involve estrogen-dependent mechanisms and can be prevented by hormonal replacement therapy [29]. In postmenopausal women, adipose tissue is a significant source of increased proinflammatory cytokines [29], which are critical mediators of bone metabolism [30,31]. However, data are not universal [21,32]. Some authors have suggested that increased fat weight, or total body weight itself, elevates musculoskeletal dynamic overloading [33]. On the other hand, others have reported the independent action of fat mass on BMD mediated by estrogen, leptin, or insulin [34,35].

## Conclusion

In postmenopausal women, low appendicular muscle mass is associated with femoral neck BMD in a short period of time. Therefore, our findings support the hypothesis that maintaining muscle mass, as age advances, attenuates femoral neck BMD loss.

## List of abbreviations

aMM: appendicular muscle mass; BMD: bone mineral density; MM: muscle mass; DXA: dual-energy X-ray absorptiometry; BMI: body mass index; SD: standard deviation; TM: time since menopause; aMM/m^**2**^: mucle mass index.

## Competing interests

Our Full Length Paper, entitled "Low appendicular muscle mass influences femoral neck bone mineral density loss in postmenopausal women", submitted to this respectable journal was carried with grants.

This investigation was supported by Fundação Lucentis de Apoio a Cultura, Ensino, Pesquisa e Extensão and Conselho Nacional de Desenvolvimento Científico e Tecnológico (CAPES). CAPES is a federal agency for the promotion of scientific and technological research and training of human resources for research in the country. The Fundação Lucentis aims to promote and stimulate the development of projects that contribute to providing better services to the community.

Futhermore there are not competing interests (political, personal, religious, ideological, academic, intellectual, commercial or any other) to declare in relation to this manuscript.

## Authors' contributions

The authors have participated sufficiently in the work to take public responsibility for appropriate portions of the content. All authors read and approved the final manuscript.

## Pre-publication history

The pre-publication history for this paper can be accessed here:

http://www.biomedcentral.com/1471-2474/12/225/prepub
